# Bacterial Secretion and the Role of Diffusive and Subdiffusive First Passage Processes

**DOI:** 10.1371/journal.pone.0041421

**Published:** 2012-08-06

**Authors:** Frank Marten, Krasimira Tsaneva-Atanasova, Luca Giuggioli

**Affiliations:** 1 Department of Engineering Mathematics, University of Bristol, Bristol, United Kingdom; 2 School of Biological Sciences, University of Bristol, Bristol, United Kingdom; 3 Bristol Centre for Complexity Sciences, University of Bristol, Bristol, United Kingdom; University of Maribor, Slovenia

## Abstract

By funneling protein effectors through needle complexes located on the cellular membrane, bacteria are able to infect host cells during type III secretion events. The spatio-temporal mechanisms through which these events occur are however not fully understood, due in part to the inherent challenges in tracking single molecules moving within an intracellular medium. As a result, theoretical predictions of secretion times are still lacking. Here we provide a model that quantifies, depending on the transport characteristics within bacterial cytoplasm, the amount of time for a protein effector to reach either of the available needle complexes. Using parameters from *Shigella flexneri* we are able to test the role that translocators might have to activate the needle complexes and offer semi-quantitative explanations of recent experimental observations.

## Introduction

The structural and rheological properties of an intracellular medium define the transport characteristics of a variety of molecules, many of which need to be transported within the cell to fulfil their tasks [1], [2]. A crucial element in carrying out these tasks is their timing and the spatio-temporal processes that control such timing. The importance of this temporal regulation cuts across a broad spectrum of cell biology; from vesicle neurotransmitters containing ribbon synapses in nerve cells [3]–[5] to neurite growth [6] and secretory activities in bacteria (see e.g. [7]). All these processes are activated as soon as a signalling agent has reached any of a set of specified locations within the cell, the so-called targets. Since the relevant dynamics is only the one that occurs before a target is reached, one talks about first-passage dynamics and defines *first-passage time* [8]–[10] as the time it takes for these triggering events to occur.

Based on a first-passage framework, here we focus on modelling secretory activities in bacteria and we provide in particular a quantitative analysis of type III secretion [11], a mechanism through which a bacterium invades potential host cells by delivering protein effectors across their membrane. The invasive process is governed by an elongated bacterial transmembrane structure, the needle complex [12], believed to represent a narrow conduit through which an effector is secreted into a host cell [13], [14]. By concentrating on the time it takes for an effector to reach a needle base, and neglecting the time to traverse the elongated needle, we provide plausible explanations of recent experimental observations on *Shigella flexneri*, a well-known bacterium which causes bacillary dysentery in humans. The experiments by [15] and [16] suggest that a significant portion of the available effector pool is depleted after several hundreds of seconds, from first contact with a host cell. Our analysis indicates that such depletion time scales are possible in either of three plausible scenarios: (1) the values of the effector diffusion coefficient are much smaller than expected [17]; (2) the effector are hampered by obstacles in the cytoplasm [18], making their movement statistics dominated by long waiting times; (3) the measured depletion time scales are the result of progressive needle activation by translocator proteins [13].

In this study we explore these three scenarios by modelling the effector motion within the bacterial cytoplasm as a random walk in a confined domain, which can escape from a narrow opening, the so-called narrow escape problem in cellular microdomains [19]. The statistical properties of this walk could be diffusive (Brownian) or sub-diffusive [20], the latter being caused by crowding effects and interactions with the complicated internal cellular structure [21]. The quantity of interest is the *mean first-passage time* (MFPT): the time that a random walker requires to arrive at a target site, averaged over all possible random trajectories. In the context of bacterial secretion it represents how long an effector protein takes on average to reach the base of a needle complex. Since bacteria, which are capable of type III secretion, often carry more than one needle complex, the MFPT needs to be averaged over multiple target sites. Computing this average is technically non-trivial since it requires distinguishing between movement paths that have reached one of the available targets without having previously reached any of the others. For the case of Brownian random walkers in circular domains, a recent study [22] has made significant advances by providing a computational tool to determine approximately a multi-target MFPT.

We exploit those advances in answering how long a single effector protein takes to reach *any* available needle complex base from a given initial position, under the assumption that it can be represented as a particle performing a random walk. Given that effector proteins are spread throughout the entire intracellular domain [15], it is difficult to identify with sufficient accuracy their initial location. We deal with this issue by studying a spatially averaged MFPT, also called the global mean first passage time (GMFPT). We examine in detail how the GMFPT depends on various parameters such as the diffusion coefficient, number of targets and target size, with parameter bounds that are mainly based on *Shigella flexneri*. Finally, we extend our modelling efforts to multiple random walkers for which we also consider the earlier mentioned needle activation by translocators. We investigate how long it takes until all walkers have escaped the domain, in two cases: ordinary diffusion as well as sub-diffusive motion.

## Results

### Models of a single effector in two and three dimensions

The average time required by one effector in the bacterial cytoplasm to reach a needle complex base depends on a number of factors. Key among them are the cell geometry, the size of the needle complex, the number and location of each complex base and the movement statistics of the protein.

**Figure 1:**
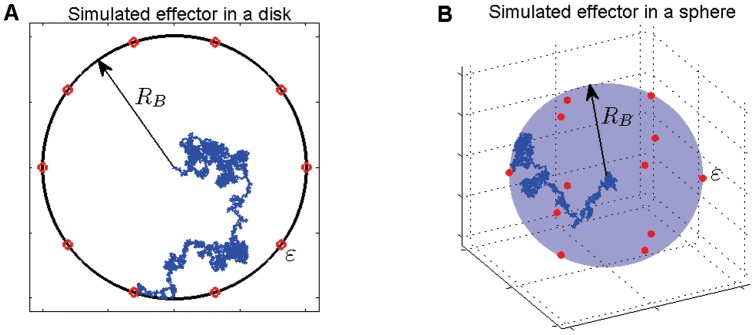
A random trajectory, representing an effector protein's movement, confined to a disk (**A**) **or sphere** (**B**) **with radius 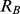.** The trajectory, assumed to be Brownian here, is shown by the blue trace. The 10 equidistant red circles in panel (A) and 12 red spheres in panel (B) are the target sites, representing the needle complex bases, whose centroids are placed on the boundary of the confining domain. We label their radius by the parameter 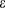.

We initially represent an effector as a Brownian particle moving within a circular domain. More specifically, we consider two different situations: the first one is a particle which performs a 2d Brownian motion in a disk-shaped domain (Fig. 1A) and the second one is a 3d motion in a sphere (Fig. 1B). When a particle reach the boundary region it is either reflected or absorbed by any of the targets on the boundary (small circular red dots in Fig. 1) and exit. The targets represent a simplified model of the actual needle complex base [12] and are shaped as disks or spheres, depending on the dimensions of the bounding domain. Each target has its centre point residing on the domain boundary and we characterize its size by the radius 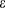 ranging from 15 to 150 Å. Moreover, the full domain size is characterized by a radius 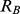 with values from 0.5 to 1.1 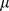
m (details about the choice of these values are given in Materials and Methods).

Experimental findings in [23] suggest that one *S. flexneri* bacterium contains at least 50 needle complexes across its membrane, similar in magnitude to the estimate for *Salmonella enterica* [24], which ranges between 10 and 100 complexes per bacterium. Measurements from electron microscopy have shown that these complexes are distributed all over the surface [25] of *S. flexneri*. Given the stochastic nature of the protein motion within the bacterium, we can represent for simplicity this random arrangement of the needles by positioning targets in a symmetric fashion on the circular boundary without significant changes in the estimation of the MFPT, but with significant computational advantages. The details on the choice of target positions, on both disk and sphere, are discussed in Materials and Methods.

### Mean first-passage time

Having defined the boundary geometry and the targets, we proceed to compute the MFPT as a function of the initial effector location. It represents how long an effector protein requires on average, from its point of synthesis within the cytoplasm, to arrive at a needle complex base. As the size of a complex base in *S. flexneri* is much smaller than the bacterium size [12], [15], it implies that 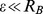. This disparity in spatial scales enables us to make use of an approximate expression for the MFPT of a Brownian particle, which has been derived in a recent work [22] for small targets on a circular boundary. Using such an expression in 2d and 3d domains with multiple targets, we are able to estimate the MFPT as function of the starting effector position.

**Figure 2:**
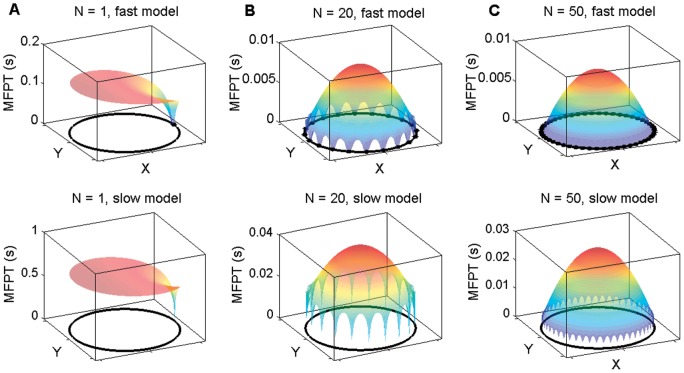
The MFPT function 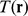 of a Brownian particle in a disk with symmetrically located targets (**as shown e.g. in Fig. 1a**). Its values are plotted as a surface which depends on the particle's starting position 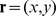 in the bottom plane of each figure. The surface colour is added only to clarify points of a large MFPT (red shading) against a small MFPT (blue shading). In the top row 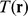 is computed for the ‘fast’ model in which 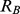  = 0.5 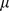
m, 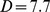 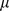
m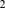/s and 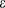 = 150 Å, with 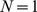, 20 and 50, correspondingly, respectively, to panel A, B and C. The bottom row shows the results for the ‘slow’ model in which 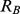  = 0.5 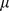
m, 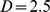 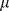
m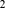/s and 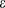 = 15 Å, and with 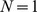, 20 and 50, correspondingly, respectively, to panel D, E and F.

For explanatory purposes, we first focus our attention on the 2d case: a single Brownian walker confined to a disk whose edge possesses a number of 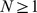 equidistant circular targets (illustrated in Fig. 1A). We fix the disk radius 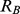 to 0.5 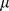
m corresponding to the size of a cross-section of *S. flexneri* perpendicular to its longitudinal axis [15]. The corresponding diffusion coefficient 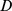 is varied from 2.5 to 7.7 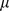
m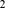/s, in agreement with fluorescence studies on protein mobility in *E. Coli* [17] (see Materials and Methods section). Accordingly, we construct two different scenarios: one ‘fast’ model for which the effector diffuses with diffusion coefficient 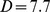 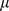
m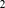/s and the target radius of the needle complexes equals 150 Å, and a ‘slow’ model for which 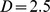 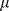
m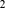/s and target radii are 15 Å. The resulting values of the MFPT, 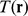, are shown in Fig. 2 as function of the effector's initial position 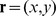, for 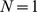, 20 and 50 targets, respectively.

From these graphs one can identify two important features of the MFPT: its dependence on the initial condition of the random walker with respect to the target locations, and the dependence on the number and size of targets. In the ‘fast’ scenario with 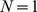 (Fig. 2A) 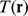 has a constant value, which goes to zero at the location of the target (blue shading), that is when the starting position is within a short distance 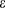 from a target center. On the other hand, if a Brownian particle starts its motion on the opposite end of the disk, with respect to the target, it will require 0.13 seconds on average to arrive at the target. In this scenario a decrease of target size and diffusion coefficient, represented by the ‘slow’ model in Fig. 2D, leads to a larger value of 0.65 seconds. The second and third column in Fig. 2 show the effect of increasing the number of needle complexes to 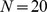 and 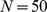, respectively. We observe that the MFPT changes to a dome shape with small arcs on the side. The highest point of this dome corresponds to the largest possible time required to arrive at any target. Clearly, a particle which starts its motion from the center of the disk will satisfy this condition. When 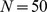 this maximum corresponds to 0.04 seconds in the ‘fast’ model, for example. The smaller arcs reveal the fact that a particle which starts close to the disk boundary between two targets has a nonzero MFPT. The height of these arcs decreases if target-to-target distance decreases to each other, as one evinces when comparing the case of 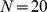 targets with the case of 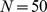 targets. As reduction in target distance for our model is analogous to an increase in the number of targets because of the symmetric placement along the circumference, to understand how 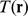 affects 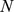 one can look sequentially at the case 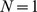, 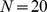 and 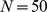 in Fig. 2. In doing that, it is clear that a variation from 1 target to 20 targets change the MFPT by one order of magnitude, whereas not much variation is observed as 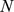 is increased to 50.

**Figure 3:**
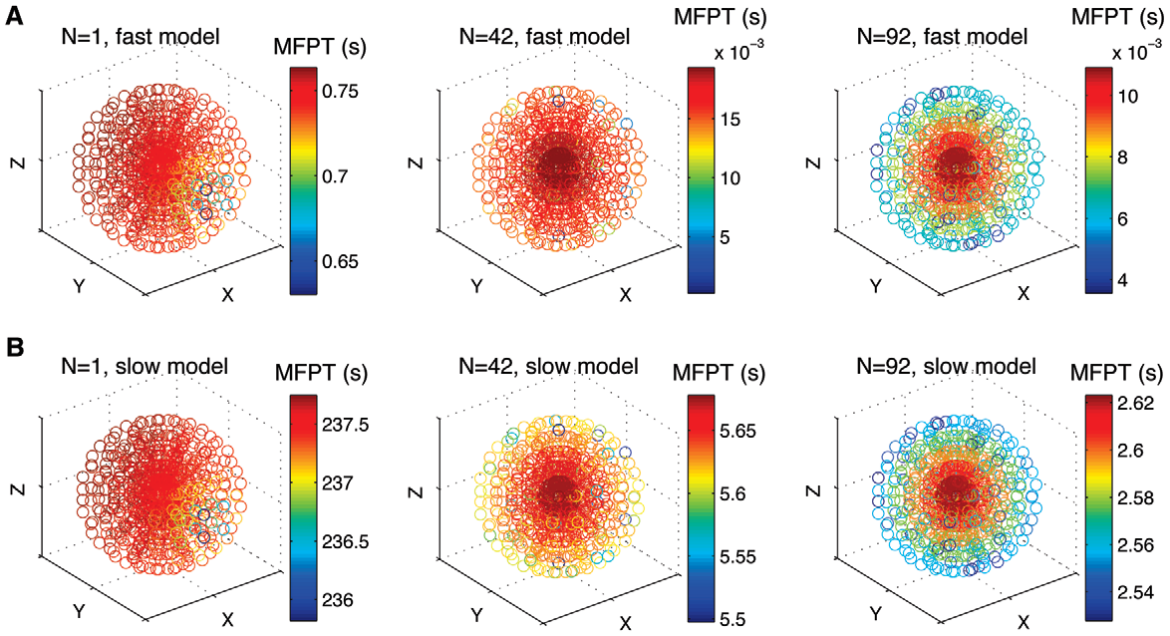
The MFPT function 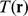 of a Brownian particle in a sphere with small spherical targets on its boundary (**not shown in the various plots**)**.** A visualization of the symmetric arrangements of the targets on the surface of the sphere has been sketched in the Materials and Methods section. The MPFT values are plotted in colour code for each subfigure as function of the particle's starting position 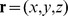 within the sphere. Starting positions are chosen in a smaller spherical region with radius 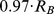 for clarity. The top row contains values of 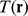 of the ‘fast’ model in which 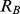  = 0.5 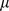
m, 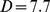 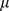
m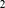/s and 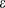 = 150 Å with 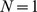, 42 and 92 targets corresponding, respectively, to panel A, B and C. The bottom row shows the results for the ‘slow’ model in which 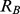  = 1.1 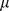
m, 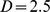 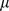
m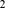/s and 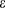 = 15 Å with 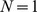, 20 and 50, corresponding, respectively, to panel D, E and F.

Similar parameter dependencies of the MFPT are observed in the 3d domain, with the needle complex bases of *S. flexneri* now modelled as spherical in shape (Fig. 1B). Similar to the 2d domain, we consider a ‘fast’ and a ‘slow’ model, the former with 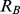  = 0.5 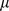
m, 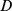 = 7.7 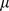
m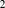/s, 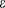 = 150 Å and the latter with 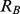  = 1.1 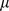
m, 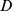 = 2.5 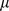
m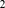/s and 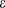 = 15 Å (see Materials and Methods for details). To represent the values of 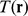 with 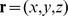, we display scatter-cloud plots in Fig. 3, for 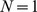, 42 or 92 targets. Each point of the cloud corresponds to an initial position of the random walker, and the colour legend reveals the corresonding value of 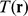.

From the top row of Fig. 3 it can be observed that a particle in the ‘fast’ model takes at most 0.76 seconds on average to reach a target – corresponding to the case when there is only one target present (Fig. 3A). In a scenario with 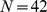 or 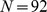, a particle which starts at the origin of the sphere takes the longest time to reach a target, compared to all other starting positions (compare red and blue colouring of the cloud dots in Fig. 3B and 3C, respectively). A visual inspection of the radial colour variation in the 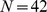 (center column) and 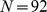 (right column) target scenarios shows that the radial gradient of 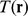 gets steeper as 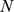 increases, which implies that a larger difference in MFPT between centered starting positions compared to those at the domain boundary, similarly to the 2d scenario. From the quantitative point of view, an effector in the ‘slow’ model has an MFPT of more than 237 seconds when only one target is present (Fig. 3D). This occurs when the particle starts near the boundary of the sphere, directly opposite to where the target is located. In the case of 42 and 92 targets, the largest MFPT value decreases considerably to 5.7 and 2.6 seconds.

In summary, if the movement statistics of an effector in *S. flexneri* were to be represented by a Brownian walker with the above mentioned diffusion coefficients, the time to reach any needle complex in 2d would be at most a fraction of a second. On the other hand, the more realistic 3d diffusion model predicts that, if only one single needle complex with a base radius of 15 Å is present, an effector protein may take up to 237 seconds on average to reach it. An increase of the target radius to 150 Å brings down the value of the MFPT to only a fraction of a second. The dependence of the MFPT on the diffusion is simple, being inversely proportional to the diffusion coefficient 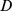. For any value 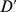 which differs from our present choice, the new MFPT can easily be derived from Figs. 2 and 3 by multiplying the colour legend values with a correction factor 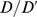.

### Spatially averaged mean first-passage time

Experimental fluorescence labeling studies in [15] revealed that effector proteins in *S. flexneri*, like IpaB and IpaC, are spread throughout the entire cell. To take into account this spatial arrangement, one needs to calculate a spatially averaged MFPT, i.e. the so-called GMFPT. If effectors are distributed according to the distribution 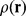, one needs to compute [22]


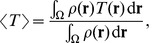
(1)

where 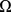 is the region where effectors are located intially. When effector proteins are equally likely to be found anywhere inside 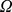, 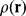 is constant and the denominator in Eq. (1) reduces to the size of the region 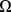. In these types of problems for which a random walker moves within a set of absorbing targets, the needle complexes, computing the GMFPT in certain cases may result in closed form expressions, e.g. with symmetric or completely random target locations [26]. As we show below the GMFPT, when targets are symmetrically distributed on the boundary, is a mathematical expressions simple enough to help understand the parameter dependence in our problem.

**Figure 4:**
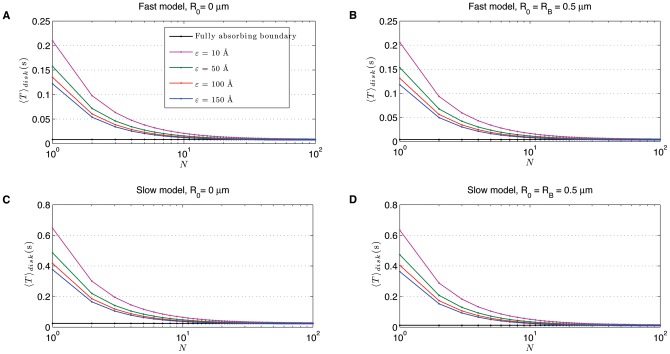
Dependence of the GMFPT 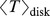, expressed in seconds, as function of the number 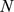 equidistant circular targets on the boundary with two different radius 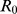 of the initial localization area 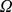: the extreme case when 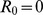 and when 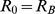, where 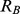 is the disk radius. Panels A and C represent the 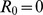 case, corresponding to an effector that starts at the origin, whereas panels B and D correspond to an initial particle localization being anywhere inside the bacterium. Each of the panels shows the GMFPT as function of 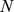 for four choices of target radius 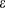 = 10, 50, 100 and 150 Å (line colour, see legend in panel A). The black curves represent a limiting case in which the entire boundary of the domain is a target; i.e. 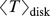 becomes the average time required to arrive at the boundary. In the top row we have considered the ‘fast’ model with 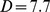 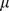
m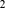/s, whereas the bottom row displays results for the ‘slow’ model with 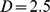 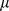
m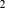/s. In all four panels 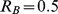 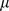
m.

As in the previous section, first we focus our attention on the 2d case: a single Brownian effector in a disk with 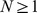 equidistant circular targets of radius 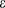. We define 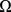 as a concentric disk-shaped region with a radius 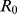 inside this domain (see a representative sketch in Materials and Methods). The GMFPT over this region is (see Supporting Information S1 for a derivation)


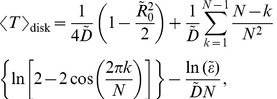
(2)

where 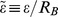, 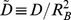 and 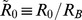. The 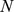-dependence of the GMFPT is dominated by the last term 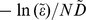 in Eq. (2), which is much larger than the term with the 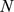-summation. For 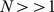 one can show numerically that this second term in Eq. (2) becomes constant. This inverse proportionality with 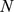 of the GMFPT can be clearly observed in Fig. 4 where 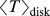 is plotted as function of 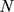 for different values of target radii 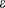 and for diffusion coefficient 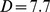 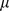
m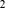/s (top panels) and diffusion coefficient 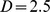 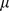
m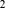/s. The left and right panels represent the two extreme cases of initial localization: with 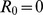 on the left and 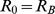 on the right, the former implying an initial condition at the center of the disk, whereas the latter implying that the initial condition could be from anywhere within the disk. Although the GMFPT in Fig. 4B and 4D changes rapidly between 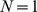 and 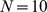 targets, if the number of targets is increased above this range, 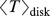 slowly decays to a limit where the entire domain boundary is a target (black curve). We also find that in the range 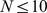 there is little difference between the values of 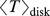 for 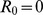 and 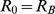 (compare Fig. 4B and Fig. 4D). Hence in a disk with less than 10 targets, the initial effector location does not play much of a role: the average time to arrive at any of the targets is roughly the same. We also notice that even in the case of one very small (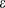 = 10 Å) target, reaching a needle complex seems to occur rather rapidly as a particle requires less than 0.7 seconds on average to reach the small target.

In the 3d spherical case with radius 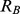 we consider that an effector can start *from anywhere* within a concentric spherical volume with radius 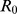 (see e.g. the figure in Materials and Methods). Performing the integration in Eq. (1) over this volume, when targets are symmetrically placed (see Supporting Information S1), we obtain


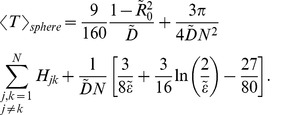
(3)

where once again 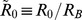, 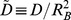 and 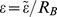. The first term of expression (3) contains the dependence of the relative size of the initial domain with respect to the size of the sphere, the terms 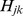 with , defined in Eq. (3.12) of the Supporting Information S1, are associated with the angular locations of the targets, and the rightmost term describes the dependence on the target size.

**Figure 5:**
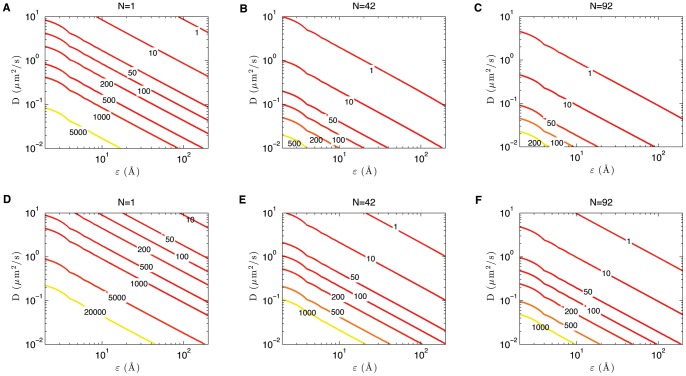
Isobars of the spatially averaged mean first passage time 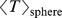 of a Brownian particle in a sphere with radius 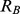 and 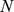 targets on the boundary, as a function of target radius 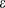 and diffusion coefficient 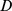. Its respective values (in seconds) along each isobar are shown by text labels. The parameter 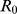 is set to 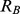 in all plots; hence we assume that the particle can start its motion from anywhere in the domain. Left to right: 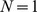, 42 and 92. Row (A): 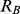 is fixed to 0.5 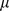
m. Row (B): 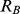  = 1.1 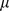
m.

By solving the associated matricial equation (see Eq. (1.1)–(1.3) in Supporting Information S1) in Fig. 5 we show values of 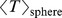 as function of target radius 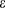 and diffusion coefficient 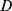 for different numbers of needle complexes (targets) and under the assumption that effectors can start from anywhere in the sphere 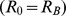. The various panels are isobars of 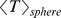 as function of target radius and diffusion coefficient for different numbers of needle complexes and two values of the bacterium radius, 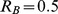 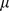
m in the top panels and 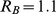 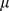
m in the bottom panels. By sequentially looking at panels A-B-C or D-E-F, it is evident that 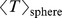 decreases as function of 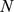. Alternatively, Fig. 5 can be used to estimate what value of diffusion constant is required to ensure a certain effector arrival time at any of the needle complexes. For example, an arrival time of 100 seconds when 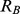  = 1.1 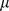
m, 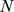 = 92 and 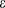 = 100 Å (Fig. 5F) requires the diffusion coefficient 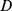 to be 0.01 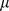
m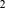/s, whereas 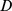 needs to be 1 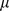
m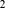/s if 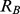  = 0.5 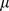
m and there is only one target of radius 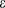 = 10 Å (Fig. 5A). For any , the relative simplicity of the expression (3) shows that to any of the values plotted in Fig. 5 one needs to rescale the result with the appropriate 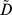 and add the quantity 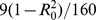.

The inverse proportionality as function of 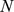 in Eq. (3) has some commonality with the 2d scenario. The term is much smaller in magnitude compared to the last term that contains the information of the radius of the targets. The 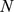 dependence is in fact controlled by the 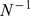 term that multiplies the square parenthesis in Eq. (3). This inverse proportionality for 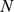 sufficiently large can be observed in Fig. 5: the countour lines shift significantly when going from 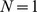 to 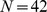 targets (compare Fig. 5A to 5B and Fig. 5D to 5E). Adding 50 more targets to the sphere, i.e. with 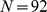 (Fig. 5C and 5B), does not change dramatically the magnitude of 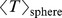. These findings imply that the average time required for an effector to reach a needle complex depends significantly on the number of such complexes only in situations for which there are a few present. On the other hand, having many (40 or more) will not alter significantly the effector arrival time at any of the needles.

### Models of effector secretion

So far we have established predictions on how much time a single protein requires to reach a needle complex base in *S. flexneri*. However, type III secretion involves many proteins, which are delivered into host cells via the needle complexes. The time scale of their escape from the bacterial cytoplasm has been visualized by fluorescence labelling studies [15], [16], demonstrating relatively slow dynamics. For instance, it takes about 240 seconds before half of the effectors in *S. flexneri* have been secreted after host cell contact [15].

Here we investigate candidate mechanisms for these slow secretion times, by focusing on a population of multiple random walkers, the effectors, moving within a bacterium of spherical shape. The effectors are assumed not to influence each other, but their movement statistics could be the result of interactions with other substances in the cytoplasm, hence they could move diffusively as well as sub-diffusively. The confining domain has 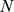 needle complex bases, which are spherical targets on its boundary. The randomly moving molecules have random starting positions within the sphere and escape from their confinement once they arrive at a target. For ease of comparison with recent experimental secretion studies [15], [16], we investigate the time dependence of the fraction of effectors left within the sphere.

We consider three scenarios: (I) diffusive effectors that escape the bacterium instantly once they reach a needle base; (II) effectors that can only escape once the needle complex has been *activated* by other types of randomly moving particles, the so-called translocator proteins [13]; and (III) proteins that move sub-diffusively rather than diffusively because of a variety of obstacles that they might encounter in the cytoplasm [18]. We define the *mean secretion time* as


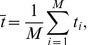
(4)

where 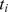 is the average time that an effector with label 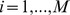 requires to escape and with the sum performed over all the 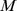 effectors. Although Eq. 4 is in general different from the MFPT computed in previous sections for a single effector, the spatially average mean secretion time in scenario (I) is however identical to the GMFPT if each protein initially is equally likely to be anywhere within a specific region of space inside the bacterium. In this case in fact 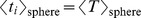 for each 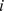, and the sum in Eq. (4) reduces simply to 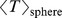. We illustrate the usefulness of this relation in the next subsection.

### I – Secretion of diffusive effectors

In this scenario we consider a population of non-interacting diffusing effectors in a sphere, escaping as soon as they reach any target site on the sphere boundary. The domain radius 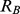 is fixed to 0.5 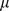
m. *Shigella flexneri* is believed to carry between 100–10,000 copies of effector proteins and we take the intermediate value of 1000 model effectors in line with effector numbers reported for Salmonella [27]. The target radii 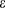 are set to 50 Å, in keeping with the needle base diameters of order 100 Å from the experimental studies in [12]. Using Eq. (4) we determine which value of diffusion coefficient 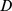 is required to give a mean secretion time 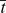 of 100 seconds when the effectors can be found anywhere inside the cell.

**Figure 6:**
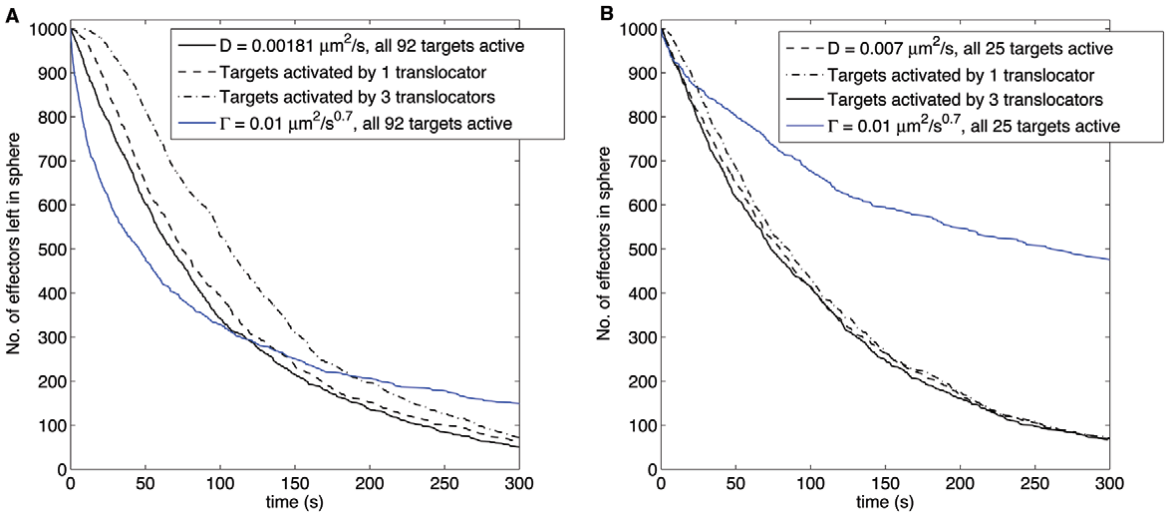
Number of effectors left in the sphere as function of time from a bacterium of spherical shape with radius 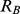  = 0.5 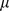
m. Panel A represents the case of 92 needle complexes distributed uniformly on the sphere. Panel B represents the case of 25 needle complexes distributed uniformly only in the upper hemisphere. In both cases the needle complexes have radius 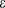 = 50 Å. The solid black curves represent the secretion of diffusive effectors (I), the dash and dash-dotted lines represent the secretion of diffusive effectors with sequentially activated needle complexes (II), and the blue line represents the secretion of subdiffusive effectors (III).

From Fig. 5F one notices that, when a cell has 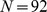 targets, a value of 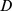 = 0.002 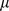
m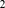/s is necessary to have 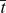 = 100 seconds. Those predictions concur with the solid black curve plotted in Fig. 6A where the number of effectors left in the sphere is drawn as a function of time. The secretion curve, generated through stochastic simulations (see Materials and Methods for the details), displays in fact an exponential decay whose mean is around 100 seconds.

Even though *S. flexneri* is estimated to carry at least 50 needle complexes [23], not all of them may be active during host secretion. Host cell contact with a specific side of the bacterium in fact leads to the formation of ‘translocation pores’ [11]. We account for such events by considering a model with only 25 needles which are distributed locally, over the upper hemisphere of the domain (see Materials and Methods). In this geometrical configuration, with needle complex radius set to 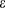 = 50 Å, a diffusion coefficient of 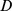 = 0.007 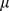
m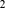/s yields an average secretion time of 100 seconds as evinced from Figure 6B, which also displays an exponential decay (solid black curve) in the number of effectors left in the bacterium as a function of time. In summary, if type III secretion consisted of effectors that move diffusively and that escaped instantly the bacterium after reaching a needle, slow diffusion with many needles and fast diffusion with only a few active needles appear to yield very similar time profiles of effector release.

### II – Secretion of diffusive effectors with sequentially activated needle complexes

The models from the previous subsection assume that every target is available as an escape channel for the effectors. However, type III secretion incorporates the assembly of a specialized translocation pore between the bacterium and its host [11]; this process requires specific translocator proteins to be present at the needle base. To take this effect into account, we simulate the secretion of effectors from the sphere under the assumption that a target becomes an active escape channel after it is visited by a translocator. If no such visit has taken place at the given target, no effectors will be able to escape through it. We add therefore at random locations inside the sphere 1000 of these translocators, which are also diffusing with the same coefficient as the effectors.

First we study a sphere with 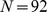 inactive targets. For comparative purposes with our earlier findings, we set 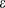 = 50 Å and 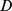 = 0.002 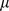
m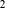/s for both effectors and translocators. If we assume that one translocator must visit a target to activate it, the dashed curve in Fig. 6A is obtained. It shows the secretion time being slightly longer when compared to the case when all needle complexes are active from the beginning (solid black curve). We have also considered the situation in which type III secretion requires three different protein types in the formation of a translocation pore [11]. For this case we consider a target active only after the arrival of three translocators. The outcome of this scenario is shown as the dash-dotted curve in Fig. 6A. Interestingly, it displays a short plateau up to 10 seconds, then falls off more steeply than the Brownian model with instantly active targets.

We also repeat the numerical simulation with 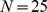 targets on the upper hemisphere with 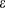 = 50 Å and 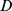 = 0.007 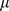
m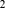/s for both translocators and effectors. Figure 6B shows the resulting secretion times. Interestingly, the effect of gradual target activation is less severe than in panel A. The average time for one translocator to meet a target is still approximately 100 seconds, as dictated by our choice of diffusion coefficient. However, the ratio of translocators/targets has increased from 1000/92 to 1000/25, and hence the chance of any of the translocator to reach either of the needle base. As a result there is a more rapid activation of the 25 targets and a greater similarity to the model with instant escape (solid black curve in Fig. 6B).

### III – Secretion of sub-diffusive effectors

**Figure 7:**
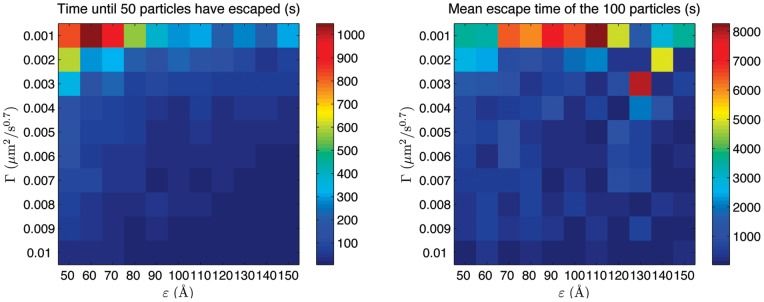
Dynamics of 100 sub-diffusive effectors escaping from a sphere with radius 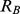  = 0.5 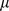
m and 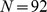 targets on the boundary. Left panel: contour plot of the time (in seconds) until the first 50 random walkers have escaped from the sphere (see legend) as a function of the target radius 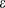 and sub-diffusion coefficient 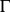. Right panel: average escape time (in seconds) of all 100 effectors (see legend) as a function of the target radius 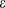 and sub-diffusion coefficient 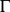.

The inside of a cell is a watery but crowded compartment with a variety of molecules and internal structures [2]. As a result, the movement of certain proteins in the cytoplasm may display kinetics slower than diffusion as observed e.g. for mRNA in the experiments by [21]. To represent this scenario in our model, we study a situation in which the effectors in the sphere also move in a sub-diffusive manner. We set the sphere radius 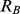 to 0.5 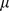
m and we place 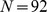 targets on the boundary. For simplicity, we assume that all needle complexes are active escape channels (no ‘translocator’ is necessary) and we put 100 sub-diffusive effectors at random locations. For each set of values of the sub-diffusion coefficient 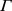 and target radius 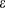 we compute two quantities: (1) the time until 50 effectors have left the sphere and (2) the average secretion time 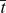 of all 100 effectors. Both quantities are displayed by a colour map, in the left and right panel of Fig. 7 respectively.

Figure 7 illustrates that, as one would expect, the sub-diffusive secretion is much slower compared to the diffusive case. The right panel shows clearly that the mean escape time of all effectors is an order of magnitude larger than the diffusive case. The left panel, on the other hand, shows that the time needed for 50 particles to leave the bacterium is comparable to secretion times of diffusive effectors. In fact, while 50 particles can leave the bacterium fairly quickly, there will be another fraction of particles which remains inside for a very long time. It is this second fraction which increases the average secretion time of the entire set of effectors.

To have a better appreciation of why the mean escape time of subdiffusive effectors may become so large, we compare secretion times of 1000 sub-diffusive effectors to 1000 diffusive effectors. When there are 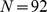 targets, the sub-diffusing effectors remain within the sphere much longer than the diffusive case (compare solid blue and black curves in Figure 6A and 6B). Whilst the first 500 of these effectors seem to escape roughly twice as fast as the equivalent 500 diffusive ones, their secretion follows a power law decay. After 800 seconds there are still roughly 90 sub-diffusing effectors left inside (not shown in the figure), and even more so when the bacterium has 25 needle complexes for which about 220 subdiffusing effectors are still inside after 10000 seconds (not shown in the figure). The rapid initial secretion of these effectors in the subdiffusive case is due to the fact that at short times the mean square displacement of an effector is 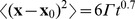, where 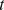 is the time variable and 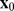 is the initial position and 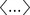 represents an average over all possible stochastic realizations. A comparison to the mean square displacement of a diffusive effector, for which 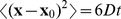, makes it clear that the different exponent in the time dependence and our choice of sub-diffusion coefficient makes a subdiffusive effector cover a larger area compared to the diffusive one at short times, and thus increasing the chance of encountering a needle complex.

**Figure 8:**
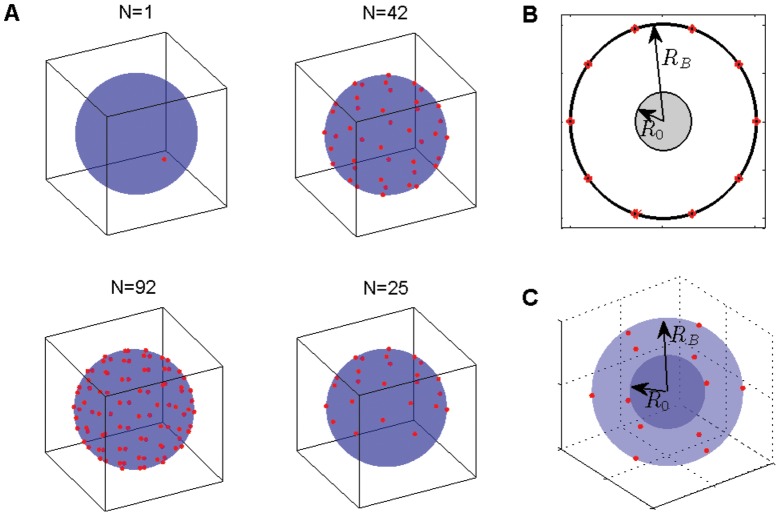
Placement of targets (**red objects**) **on the boundary of a sphere.** In the top left corner of panel (A), a single target is displayed. In the top right and bottom left corner of panel (A) 42 and 92 targets are placed according to a geodesic grid. In the bottom right corner of panel (A), the geodesic grid for 25 targets is used but only to half of the sphere. The fourth panel shows 25 targets on the upper hemisphere – to study localized activation of needle complexes. (B) If we have no information about the starting location of a random walker in a disk, we assume that it is uniformly random within a smaller disk of radius 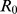. (C) The same idea applied to a random walker in a sphere; we assume that it can start anywhere within a smaller spherical volume of radius 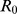.

## Discussion

We have developed a mathematical model to represent the movement of a particle moving randomly within a circular microdomain with one or more small openings. The model provides predictions for the average time a particle takes to reach either of the available openings based on the number of such openings, the size of the microdomain and the particle movement statistics. We have used the mathematical formalism to investigate bacterial secretion in a rigorous quantitative way. As a model study we have considered the movement of protein effectors within *Shigella flexneri* with the goal to offer testable hypotheses on the mechanisms that regulate the timing of type III secretion. The derived mathematical expressions can in fact be used to interpret secretion processes in other type III secretion systems such as *Escherichia Coli* [28] and *Salmonella* [29], [30].

**Table 1:** Parameter ranges in our (sub)diffusive models.

Name	Short description	Value & units
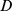	Diffusion coefficient	2.5 to 7.7 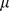 m 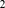 /s
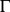	Subdiffusion coefficient	10 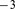 to 10 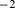 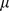 m 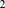 /s 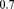
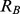	Radius of confining domain (disk or sphere)	0.5 or 1.1 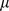 m
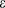	Radius of targets	15 to 150 Å
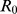	Radius of initial effector locations	0 to 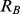

Throughout our analysis we have assumed that the movement of an effector inside *S. flexneri* is that of a random walk constrained within a circular domain, whose movement statistics is either diffusive or sub-diffusive. The domain boundary contains small circular objects which serve as models of a needle complex base. We have employed this framework for two purposes: (1) to predict the average time that one effector requires to reach a needle base; (2) to probe how the secretion time of multiple effectors is influenced by factors such as needle complex size, number of needles and diffusion or subdiffusion coefficients.

In order to achieve (1), we have computed the MFPT of an effector to any needle base in two different circular domains; a disk and a sphere. Under the assumption of diffusive motion, we have found that the MFPT depends strongly on the number of needle complexes when only a few needles are present. For example, a diffusive effector with a coefficient of D = 2.5 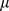
m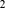/s, in a sphere, takes about 237 seconds to reach one single needle complex with a radius of 15 Å. If 42 of such complexes are present on the sphere boundary, the average time to reach any of them reduces to merely 5 seconds. An increase to 92 complexes only halves the time to 2.6 seconds. This variation in time scales holds for other choices of 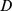 and needle radii as well. We have used these kinds of quantitative predictions to estimate how many needle complexes are activated in *S. flexneri* during host cell contact. By comparing our findings to experimental observations we find that to account for secretion times in the range 100–1000 seconds [15], our model suggests that either few needle complexes are active if the diffusion coefficient 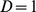 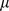
m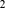/s or many needles are active, but 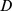 needs to be a few order of magnitudes lower than 1 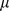
m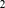/s.

Given the uncertainty about the initial protein locations, we have developed a formalism to take into account that effectors may be spread out throughout the entire bacterium. We have derived general analytic expressions for the GMFPT, that is the spatially averaged MFPT, whose validity has been tested with detailed stochastic simulations. From the mathematical equations it is straightforward to determine the dependence of the GMFPT as function of the number and size of the needle complexes as well as the diffusion constant of the effectors and their initial locations. The results of our analysis, although focused on protein secretion in a particular bacterium, is general enough to be used as a tool to estimate arrival times in other narrow escape problems.

Our 3d model of diffusive effectors in *S. flexneri* predicts that, in case of only a few active needles, the initial location of an effector does not significantly influence its arrival time at a needle base, unless this location is very close to a target. Conversely, if many active needles are present, an effector which starts its motion in the center of the sphere takes twice as long to reach a needle; when compared to one that starts near the boundary. Another spatial heterogeneity that may affect our estimates is the initial localization of the effectors as reported by [31]. Although the full implications on secretion times of heterogenous placement of needles and effectors would require an extensive study, one may estimate their effects by using our results. The slowest scenario would correspond to the generation of effectors at one pole while the needle complexes are concentrated around one location on the opposite side. This would be the only situation which might give MFPT times of the order of 200 seconds (see Fig. 3D for the case of a single needle complex). If, however, multiple needle complexes are evenly distributed throughout the bacterium as reported in [25], the MFPT for each effector gets reduced to 5 seconds (see Fig. 3B for the case of 42 needle complexes).

In presence of multiple effectors we have defined a mean secretion time and we have limited our analysis to the 3d spherical case. We have studied the dependence of secretion times as function of the number of needle complexes and the effector movement statistics, diffusive and sub-diffusive. We have simulated the escape times of 1000 randomly moving effectors, which leave the bacterium as they reach one of the targets on its surface. Recent experimental studies [15], [16] have provided measures of the effector concentration, and its decrease, in *S. flexneri* after host cell contact. For comparative purposes, we have plotted the number of effectors which are still in the sphere as a function of time. The shape of these simulated secretion curves strongly depends on the particular model taken: diffusive effectors which escape the sphere after reaching a target, display an exponential decay. If targets are gradually activated by randomly moving translocators, the effector secretion curve displays a moderate plateau for short times, and then falls off more steeply than the earlier mentioned exponential trends. Lastly, we have analyzed the secretion dynamics of 1000 sub-diffusive effectors. We have observed that the first 500 leave the sphere faster than previous cases. This peculiarity is because the average area that a sub-diffusive particle explores at short times is larger than the average area covered by diffusing particles. But more importantly, we have observed that the remaining effectors stay within the bacterium for 1000 seconds or more.

Some of our model findings are in agreement with recent experimental observations [15], [16]. For example, Fig. 14.2 in [16] reveals a moderate secretion up to 120 seconds, followed by a much steeper drop in effector concentration past this time – similar to the plateau shape in the dash-dotted curve of our Fig. 6A for times less than 10 seconds. Analogous trends can be seen in Fig. 5 in [15]. Hence our model predicts that, if effector motion were to be diffusive, the initial segment of the secretion curves in *S. flexneri* is shaped by the *number of translocators* that are required to activate a needle complex. In relation to the movement statistics of the effectors, it appears that sub-diffusion, that is random displacements with extremely long, albeit rare, waiting times, may not be the most adequate model to represent effector protein motion. Our model with subdiffusing effectors yields in fact secretion curves that decay as power law at long times. This means that a significant fraction of effectors is still present in the confining micro-domain after more than 1000 seconds from simulation onset. The experimental measures in [15] do not exhibit such trends, making this a less plausible scenario, unless the experimental measurements are not able to detect effectors within the bacterium when their number is small.

In the interest of simplicity, we have not attempted a more detailed description of what takes place at the base of a needle complex e.g. how translocator proteins assemble a secretion channel into a host cell, or how effectors pass through such channels. If these processes do not generate statistical correlations between the movement trajectories of the individual molecules, one can estimate secretion times by adding a fixed amount to the first passage time expressions presented here. If, on the other hand, the molecules develop exclusive interactions (see e.g. [32] as an example of exclusion dynamics in biology) because of their non-negligible size, queuing effects become important and spatio-temporal correlations in the molecule trajectories may emerge. In such scenarios the effector release time may increase even further and a detail modelling of these mechanisms become necessary if a rigorous quantification is seeked.

## Materials and Methods

### Random walks in a circular confining domain

The movement of the effector proteins inside *S. flexneri* is modeled by (1) diffusive and (2) sub-diffusive motion. The effector protein is represented by a random walker that roams within the bacterium, represented by a disk or a sphere with radius 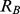 as shown in Fig. 1. The domain boundary, in the absence of any target, is reflecting: every attempt to move past the boundary simply results in a reset of the walker to its last position within the domain. The movement of each effector is computed by selecting a displacement vector and a waiting time that represents the elapsed time before the actual movement of the effector occurs. For the diffusive case the position 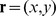 in 2d or 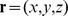 in 3d of an effector is updated by drawing spatial increments chosen, respectively, from a 2d or 3d Gaussian distribution, and the waiting times from an exponential distribution. For the sub-diffusive case, the difference is in the selection of waiting times, which are drawn from a distribution, which has a power-law tail [33].

An effector keeps moving inside the bacterium until it reaches one of the absorbing targets. The targets, representing the intracellular base of the needle complexes in *S. flexneri*, are modelled as small circular or spherical objects with radius 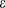. As the time it takes to move from the base to the tip of the needle complex is considered negligible in our model, the first passage time for an effector to be secreted takes into account only the dynamics to reach the base of a target. The midpoint of these targets is placed exactly on the domain boundary, as shown by the red circular objects in Fig. 8. Electron microscopy imaging studies [25] suggest that needle complexes are distributed over most of the cellular surface. For simplicity we place 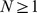 targets to cover the boundary domain in a uniform way. For the disk we simply choose an equidistant placement (see e.g. Fig. 8B). In 3d it is accomplished by constructing a geodesic grid, that is by distributing initially a subset of the targets as the vertices of an icosahedron tangent with the sphere, adding the additional ones so that they are equidistant between the vertices of the icosahedron and then projecting all the 3d target locations onto the sphere.

To understand how results change in *S. flexneri* as function of 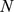, we have studied in particular the cases with 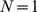, 42 and 92 shown, respectively, in the top left, top right and bottom left of Fig. 8A. To capture *S. flexneri* cells which activate only a fraction of their needle complexes, near the surface of a host-cell [11], we also study the special case where 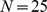 targets are placed on the upper hemisphere (see the bottom right panel of Fig. 8A).

### Parameter values from experimental studies

The molecular mass of proteins that are involved in type III secretion by *S. flexneri* spans a range of values from 38 kDA (IpaD) to 70 kDa (IpaB) as reported in [34]. An experimental study by [17] considered fluorescently labeled proteins with similar mass moving within the *E. Coli* cytoplasm. By assuming that proteins diffuse, a diffusion coefficient *D* = 2.5 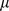
m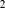/s is estimated for heavy 72 kDa proteins, whilst for lighter proteins of around 28 kDA gives *D* = 7.7 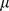
m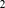/s. We have used values in the above range for the effector diffusion coefficient when estimating MFPT and GMFPT to any of the needle complex bases. In the study of secretion times, in order to reproduce certain quantitative features of the experimental observations, we have used diffusion coefficient values between 50 and 200 smaller than values one would expect from similar size molecules in *E.Coli* studies.

Large molecules, such as mRNA, may be hindered in their motion through the bacterial cytoplasm because of overcrwoding effects [21]. As a consequence their motion is slowed considerably. This slowing down can be mimicked by making the effectors wait much longer between subsequent spatial displacements. For this subdiffusive aspect of the motion a generalized diffusion coefficient 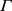 ranging from 0.001 to 0.01 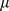
m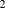/s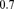 has been used, which is in the range of mRNA motion in *E. Coli* cytoplasm [21].

The shape of *S. flexneri* is elongated with a length around 3 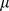
m. A circular cross section, perpendicular to its longitudinal axis, has a diameter of about 1 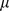
m [15]. If one were to consider the elongated shape to be a cylinder, its volume would be 2.4 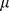
m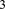. A sphere with the same volume has a radius of 0.82 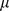
m. Given a variability between 0.5 to 5 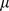
m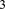 observed in the volume of *E. Coli* [35], we consider a similar range in our study. A sphere with equivalent volume has radii of about 0.5 and 1.1 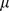
m, respectively. These two values are the ones used for 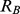 in our investigations.

A needle complex has an internal diameter of about 30 Å [16]. The transmembrane region of the complex, i.e. the base which is embedded in the intracellular membrane, has a diameter of 300 Å [12]. For analysis, we cover that range of values by using target radius 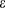 between 15 Å and 150 Å. A full summary of each parameter is provided in Table 1.

### Mean first-passage time models and errors

In the diffusive case to compute efficiently the mean first-passage time 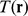 of an effector protein to reach a needle complex base (Figs. 2 and 3), we have made use of the approximate results from [22] for both the 2d and 3d scenario. For convenience the mathematical expression for 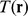 has been reported in the Supporting Information. Although those expressions are valid for sufficiently small target radii with 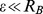, an estimate for the error in estimating 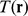 is provided as follows. For a disk with 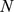 targets it is given by [22]


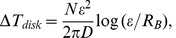
(5)

and for a sphere


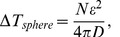
(6)

In both cases, the error functions increase as 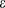 and 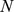 increase.

Let us consider some illustrative cases in 2d. The first case is a diffusive walker in a disk with 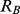  = 0.5 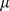
m, 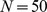 targets and 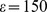 Å, as shown in Fig. 2C. A diffusion coefficient 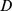 = 7.7 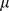
m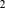/s leads to an error of order 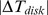  = 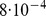 seconds. This is around 8% of the value at the peak of 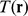. The second case is a disk with 50 smaller targets of 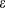 = 15 Å and 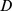 = 2.5 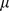
m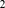/s, as shown in Fig. 2F. The corresponding error is 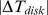  = 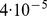 seconds. This is about 0.1% of the peak of 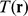.

We also compute 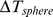 in case of 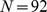 targets on a sphere. For 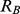  = 0.5 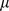
m and 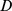 = 7.7 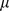
m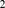/s, as in Fig. 3C, we find that 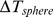  = 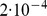 seconds. This is about 2% of the value of 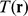 for a random walker which starts in the center of the sphere (red legend colour). If the random walker were to start near the edge of the sphere (blue legend colour) the error would be 5%. As shown in the Supporting Information S1, we determine the accuracy of 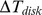 and 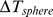 evaluated through Eq. 5 and 6 by calculating 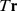 from stochastic simulations. We find that 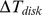 is actually only a few percent of the peak value of 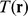 for 50 targets, whereas 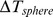 is only a sub-percentage of the maximum of 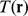 for 92 targets. This error analysis confirms the validity in estimating 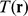 using Eq. (2) and (3).

### Spatially averaged mean first-passage time in a sphere

The terms 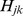 are a shorthand notation for the so-called pseudo-Green's function 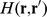 which is evaluated at the centerpoints 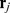 and 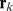 of targets with label 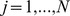 and 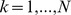 respectively. For a Brownian walker such functions can be found in [36] and for convenience have been rewritten from [22] in the Supporting Information S1.

## Supporting Information

Supporting Information S1 Details of the mean-first passage time calculations together with a comparison to stochastic simulations.
(PDF)
