# Supplementary material for: Bacterial Secretion and the Role of Diffusive and Subdiffusive First Passage Processes
Source: PLoS One. 2012 Aug 6;7(8):e41421. doi: 10.1371/journal.pone.0041421 (PMC3412870; doi:10.1371/journal.pone.0041421)
Supplement: Supporting Information S1 — Details of the mean-first passage time calculations together with a comparison to stochastic simulations. (PDF) [file pone.0041421.s001.pdf]

Supporting Information S1
F. Marten, K. Tsaneva-Atanasova, L. Giuggioli
Bacterial secretion and the role of diffusive and subdiffusive first passage processes

1 Calculations of the mean first passage time

Various quantities in our work are based upon the model by Chevalier *et al.* [2]. Here we briefly discuss the key components of that approach and we provide details of how we have used their mathematical development to obtain mean first passage time (MFPT) expressions, as well as how we have tested the accuracy of such expressions.

The needle complexes are represented by small circular (2d) and spherical (3d) targets with radius ε and center positions on the boundary of the confining regions, respectively, a circle in 2d and a sphere in 3d. An effector is represented as a random walker that moves either diffusively or subdiffusively, and gets absorbed when it hits one of the available targets. If the particle hits any other part of the boundary, it gets reflected inside. In presence of just one target one needs to compute the first passage probability to reach the target, from which one can deduce the mean first passage time to the target. Here, however, the situation is more complicated due to the presence of multiple targets. In this case one needs to compute splitting probabilities $P_1(\mathbf{r})...P_N(\mathbf{r})$ for each of the N targets. For illustrative purpose in Fig. 1a we show a sample trajectory of an effector that starts at the origin and hits one of the $N = 20$ available targets on the boundary of a disk. In Fig. 1b we display the splitting probability associated with the specific target at coordinates ($x = 1$,$y = 0$) as function of the initial effector location. In Fig. 1c we plot the MFPT with $N = 20$ needle complexes as function of the initial effector position.

In general terms given a confined domain the splitting probability $P_i(\mathbf{r})$ represents the chance that, for a particle starting its movement at position $\mathbf{r}$, a target with label i will eventually be hit without visits to any other targets beforehand. When the movement of the effectors is diffusive, i.e. when the random walkers are Brownian, the splitting probabilities for both the unit disk and the unit sphere, $\mathbf{P}(\mathbf{r}) = [P_1(\mathbf{r})...P_N(\mathbf{r})]^T$, satisfy the following linear algebraic system [2]:

$$M\mathbf{P}(\mathbf{r}) = \mathbf{b}(\mathbf{r}), \tag{1.1}$$

where the matrix M is defined as

$$M_{ij} = 1 + \sum_{k=1}^{N} \frac{H_{ij} - H_{jk}}{H_{kk}} \qquad i, j = 1, ..., N, \tag{1.2}$$

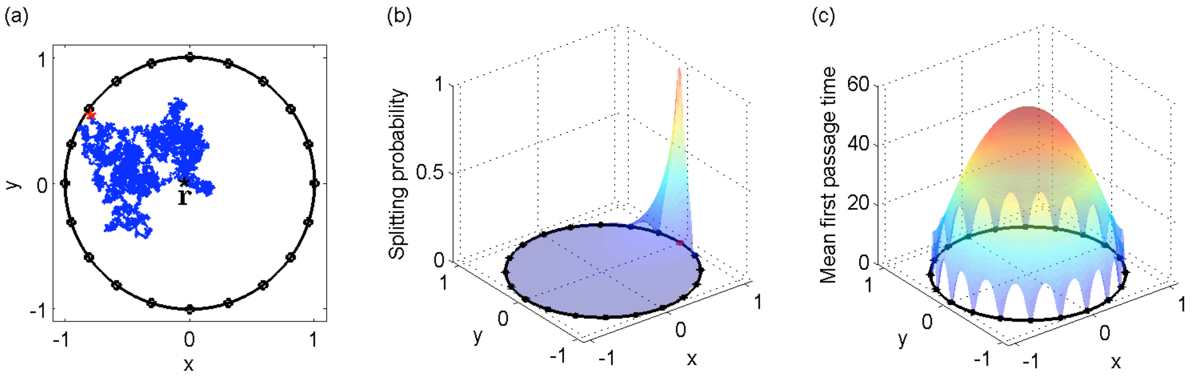

Figure 1: Unit disk with $N = 20$ symmetrically placed targets. (a) Sample Brownian walk trajectory to reach one of the absorbing targets. The black star represents the starting position whereas the red star is the final location before absorbtion occurs. (b) Splitting probability P_1 for the target located at $(x = 1, y = 0)$ as function of the initial walker position. The other 19 splitting probability function P_2-P_{20} are not shown. Panel (c) displays the MFPT as a function of a Brownian walker's intial position. The color coding is present only for visual help.

and the elements of the column vector $\mathbf{b}(\mathbf{r})$ are

$$b_i(\mathbf{r}) = 1 + \sum_{k=1}^{N} \frac{H_{iS} - H_{kS}}{H_{kk}} \qquad i, j = 1, ..., N. \tag{1.3}$$

The expressions for H_{ij} and H_{kS} are compact notations for the pseudo-Green's function [1] $H(\mathbf{r}_i|\mathbf{r}_j)$ in which $\mathbf{r}_i$ and $\mathbf{r}_j$ are the center positions of the absorbing i-th and j-th target, respectively. On the other hand, the symbol S in the expression H_{kS} is a short-hand notation for $H(\mathbf{r}_k|\mathbf{r})$ with $\mathbf{r}$ the initial starting location of the effector and $\mathbf{r}_k$ the center of the k-th absorbing target. When $\mathbf{r} \neq \mathbf{r'}$ the H function in the unit disk case is given by

$$H(\mathbf{r}|\mathbf{r'}) = \frac{1}{2\pi}\left\{-\ln|\mathbf{r}-\mathbf{r'}| - \ln\left|r'\mathbf{r} - \frac{\mathbf{r'}}{r'}\right| + \frac{r^2 + r'^2}{2} - \frac{3}{4}\right\}, \tag{1.4}$$

whereas when $\mathbf{r} = \mathbf{r'}$ and $|\mathbf{r}| = 1$ one has

$$H(\mathbf{r}|\mathbf{r}) = -\frac{1}{\pi}\ln(\varepsilon) + \frac{1}{8\pi}, \tag{1.5}$$

Eq. (1.4) allows us to evaluate the terms H_{mn} with $m \neq n$ and H_{nS} in Eq (1.2) and (1.3), whereas we can compute H_{nn} for the targets on the boundary of a disk by using Eq. (1.5).

When $\mathbf{r} \neq \mathbf{r'}$ the H function in the unit sphere case is given by

$$H(\mathbf{r}|\mathbf{r'}) = \frac{1}{4\pi|\mathbf{r}-\mathbf{r'}|} + \frac{1}{4\pi|\mathbf{r} - r^2\mathbf{r'}|} + \frac{1}{4\pi}\ln\left[\frac{2r}{r - r(\mathbf{r}\cdot\mathbf{r'}) + |\mathbf{r} - r^2\mathbf{r'}|}\right] + \frac{r^2 + r'^2}{8\pi} - \frac{7}{10\pi}, \tag{1.6}$$

whereas when $\mathbf{r} = \mathbf{r'}$ and $|\mathbf{r}| = 1$ one has

$$H(\mathbf{r}|\mathbf{r}) = \frac{1}{2\pi\varepsilon} + \frac{1}{4\pi}\ln\left(\frac{2}{\varepsilon}\right) - \frac{9}{20\pi}. \tag{1.7}$$

Similarly to the 2d case, Eq. (1.6) allows us to evaluate the terms H_{mn} with $m \neq n$ and H_{nS} in Eq (1.2) and (1.3), whereas we can compute H_{nn} for the targets on the boundary of a sphere by using Eq. (1.7).

From the knowlegde of $\mathbf{P}(\mathbf{r})$ it is possible to obtain an expression for the MFPT of the Brownian particle [2]:

$$T(\mathbf{r}) = \frac{V}{D \sum_{k=1}^{N} \frac{1}{H_{kk}}} \left(\sum_{k=1}^{N} \sum_{j=1}^{N} P_j(\mathbf{r}) \frac{H_{jk}}{H_{kk}} - \sum_{k=1}^{N} \frac{H_{kS}}{H_{kk}} \right) \tag{1.8}$$

where V is the generalized volume of the confining region (in the unit disk it is π, in the unit sphere it is $3\pi/4$) and D is the diffusion coefficient. Equation (1.8) gives the average time for a Brownian particle, which starts its motion at $\mathbf{r}$, to be absorbed by *any* of the N targets for the first time. Alternatively, it is the average time after which a Brownian particle hits one of the targets for the first time.

All the above expressions are detailed in Chevalier *et al.* [2], but we have written them out explicitly here for their use on the following sections. Furthermore, all the expressions that appear in this document are displayed, for simplicity, for the unit disk and sphere. When the confining region has radius R_B, one can rescale the problem to one inside the unit disk or sphere by simply using the relations

$$\mathbf{r} \to \mathbf{r}/R_B, \quad \varepsilon \to \varepsilon/R_B, \quad D \to D/R_B^2 \tag{1.9}$$

for, respectively, the target or effector initial location $\mathbf{r}$, the target radius ε and the effector diffusion coefficient D. Note that since the original D has dimensions of μm^2/s, its rescaled version will have units of s^{-1}. Equation (1.8) then gives the desired first-passage time in units of s.

2 Accuracy of the MFPT model

The expression in Eq. (1.8) is derived via a perturbation analysis approach [2], taking into account the limit of a small but nonzero target radius $\varepsilon \ll 1$. The estimate associated with $T(\mathbf{r})$ is obtained by computing [2]

$$\Delta T \simeq \frac{N\varepsilon^d}{D} G_0(\varepsilon), \tag{2.1}$$

where d is the spatial dimension of the problem ($d = 2$ in the unit disk and $d = 3$ in the unit sphere) and N is the number of targets. $G_0(\varepsilon)$ is the singular part of a pseudo-Green's function and is given by $G_0(\varepsilon) = -(2\pi)^{-1} \ln(\varepsilon)$ and $G_0(\varepsilon) = (4\pi\varepsilon)^{-1}$ for the unit disk and unit sphere, respectively. For any $\varepsilon > 0$, ΔT decreases if the radius of the absorbing targets decreases.

Although in the main text to estimate the error associated with the approximate value of the MFPT in expression (1.8) we have made use of Eq. (2.1), we have not discussed the validity of the expression for ΔT. Here we do that by computing the magnitude of T from stochastic simulations and comparing it to the value for T obtained via Eq. (1.8). For this purpose we consider a Brownian particle in a sphere or disk of radius $R_B = 0.5$ μm with N absorbing targets. We select the so-called "fast model", corresponding to a choice of diffusion

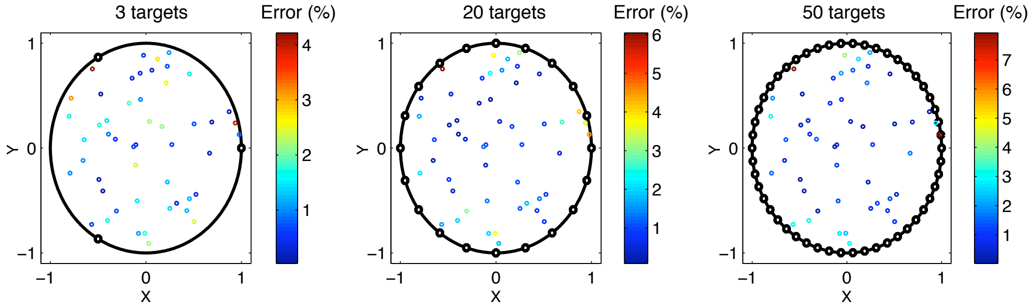

Figure 2: Calculation of the error of the analytic expression for the MFPT for a Brownian walker starting at different locations inside the disk for the "fast model", that is with an effector diffusion coefficient $D = 7.7\ \mu^2$/s, a disk radius $R_B = 0.5\ \mu$ and target radii equal to 150 Å. From left to right the panel represent disks with $N = 3$, 20 50 targets. The error is calculated by computing $\left|\frac{T-T_{simul}}{T}\right| \times 100\%$ where T_{simul} is the MFPT obtained by averaging over 10,000 simulated trajectories and T is obtained from Eq. 1.8. Each colored point inside a disk represent the initial location of the walker and the magnitude of the error is given by the color-coding to the right of each panel.

coefficient $D = 7.7\ \mu\text{m}^2$/s and target radii equal to 150 Å in 2d and equal to 200 Å in 3d as described in Sec. 2 of the main text.

For the disk we have carried out this comparison for the "fast model" when the number of absorbing targets is $N = 3$, 20 and 50. The spatial dependence of the MFPT in Eq. (1.8) when $N = 20$ and 50 are displayed in Fig. 2C of the main text. Direct application of Eq. (2.1) for a Brownian walker with starting position in the middle of the disk yields an error estimate of $\Delta T = 8 \cdot 10^{-4}$ s, corresponding to an 8% error. This error estimated from Eq. (2.1) becomes larger than 95% for starting locations near the disk boundary. By choosing a variance in our Brownian motion integrator for the simulated trajectories such that $\sqrt{2D\Delta t} = \varepsilon/15$, one obtains error which are smaller than those predicted by Eq. (2.1). For example in the case $N = 50$, when an effector starts at the center of the disk, the expression (1.8) of the MFPT is off by an error of the order of 1%, whereas it becomes of the order of 10% when the effector initial condition is close to the boundary. If the number of targets are reduced to $N = 3$ the error decreases even further. These values and its dependence on the initial walker position are displayed in Fig. 2 by calculating $\left|\frac{T-T_{simul}}{T}\right| \times 100\%$, where T_{simul} is the value obtained from the simulated trajectories, and expressing it in percentage value.

A similar analysis has been performed for the sphere but with the choice of N being 12, 40 and 92 absorbing targets. The largest errors are typically found for initial conditions close to the boundary and in proximity of absorbing targets and are of the order of 7%. In this 3d scenario we have also looked at the effects due to a change in the choice of the variance of our Brownian motion integrator. For example for the case with $N = 92$, if we set $\sqrt{2D\Delta t} = \varepsilon/15$, that is, 15 times smaller than the absorbing target radius, the largest error is around 7% for those starting locations closer to the targets (see right bottom panel in Fig. 3). If $\sqrt{2D\Delta t}$ is decreased even further, to $\varepsilon/60$, this error decreases to 0.9%. Similar reduction in the error bounds are also found for the other choices of N.

In summary Eq. (2.1) for our system provides an overestimation of the error and we can

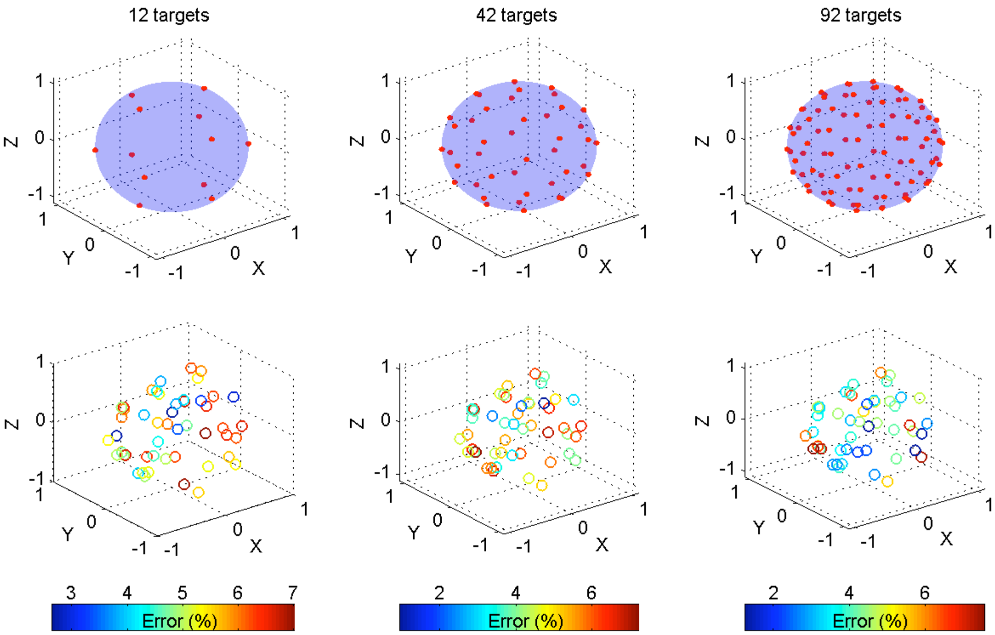

Figure 3: Calculation of the error of the analytic expression for the MFPT for a Brownian walker starting at different locations inside the disk for the "fast model", that is with an effector diffusion coefficient $D = 7.7\ \mu^2/\mathrm{s}$, a disk radius $R_B = 0.5\ \mu$ and target radii equal to 200 Å. All top panels show the number and locations of the N targets with $N = 12$, 40 and 92 in moving from left to right. The error is calculated by computing $\left|\frac{T-T_{simul}}{T}\right| \times 100\%$ where T_{simul} is the MFPT obtained by averaging over 10,000 simulated trajectories and T is obtained from Eq. 1.8. For all the bottom panels, each colored circle represents the initial location of the walker and the magnitude of the error can be deduced with the help of the color-coding at the bottom of each panel.

thus reliably use Eq. (1.8) to estimate the MFPT. We have been able to show that in 2d Eq. (1.8) with $N \leq 50$ gives results which are accurate between 1% and 10%, whereas in 3d Eq. (1.8) is accurate to 1% or better when $N \leq 92$.

3 Spatial averaging

To make use of Eq. (1.8) for our experimental situation, it is necessary to determine the initial position $\mathbf{r}$ of an effector. Unfortunately this information is not readily available and one either assumes that an effector is equally likely to be synthesized anywhere inside the cell or that it is synthesized only within certain regions inside the cell. To take into account this uncertainty one needs to modify the MFPT expression by integrating $T(\mathbf{r})$ in Eq. (1.8) over an area within the unit disk or a volume within the unit sphere. In other words one needs to calculate

$$\langle T \rangle = \frac{1}{\Omega} \int_\Omega T(\mathbf{r})\, \mathrm{d}\mathbf{r}, \tag{3.1}$$

where the symbol $\langle ... \rangle$ means that a spatial average has been performed, and where Ω is the size of the region where it is equally likely to find an effector. This quantity is called the *global mean first passage time* (GMFPT).

A close inspection of Eq. (1.8) reveals that there are only two terms in $T(\mathbf{r})$, which depend explicitly on the initial starting location $\mathbf{r}$, namely the sum containing $P_j(\mathbf{r})$ and the pseudo-Green's function $H_{kS} = H(\mathbf{r}_k|\mathbf{r})$. The GMFPT expression in Eq. (3.1) is thus given by

$$\langle T\rangle = \frac{V}{D\sum_{k=1}^{N}\frac{1}{H_{kk}}}\left(\sum_{k=1}^{N}\sum_{j=1}^{N}\langle P_j\rangle\frac{H_{jk}}{H_{kk}} - \sum_{k=1}^{N}\frac{\langle H_{kS}\rangle}{H_{kk}}\right), \tag{3.2}$$

where $\langle P_j\rangle = \Omega^{-1}\int_\Omega P_j(\mathbf{r})\,\mathrm{d}\mathbf{r}$ and $\langle H_{kS}\rangle = \Omega^{-1}\int_\Omega H(\mathbf{r}_k|\mathbf{r})\,\mathrm{d}\mathbf{r}$.

When the targets are placed in a symmetric fashion on the confining boundary, the integration in Eq. (3.1) can be done analytically. In the following we explicitly show how to obtain the simplified analytic expressions for the GMFPT in those symmetric cases both in 2 and 3d.

3.1 Spatial averaging in 2d

For the unit disk, we consider the region where effectors may be located as a concentric circle A with the dimensionless radius R_0 (see Fig. 4a). In this case the spatial average in Eq. (3.1) is given by

$$\langle T\rangle_{\text{disk}} = \frac{1}{\pi R_0^2}\int_0^{R_0}\int_0^{2\pi} T(r,\theta) r\,\mathrm{d}\theta\mathrm{d}r, \tag{3.3}$$

where the integral is performed over the polar coordinates (r,θ).

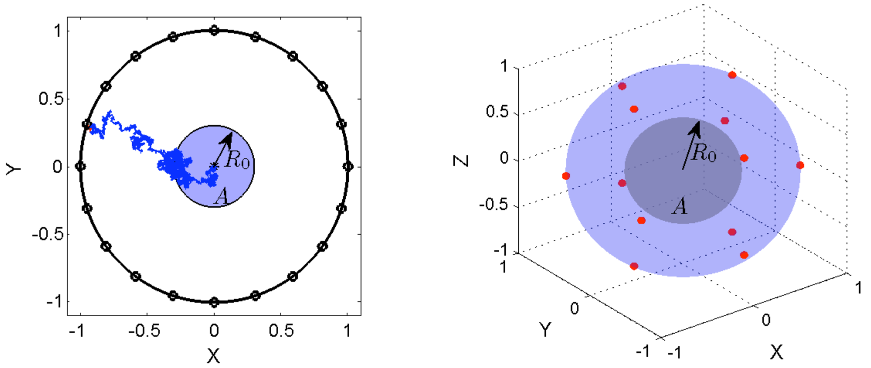

Figure 4: Choice of the region of integration for Eq. (3.3). Left panel: A is the region of space with radius R_0 in a disk within which a Brownian walker can be found initially with uniform probability. The $N = 20$ black circles on the circumference of the disk represent the absorbing targets. The dark blue jiggling line illustrates a sample trajectory of a Brownian particle starting at the center of the disk. Right panel: A represents the region of space with radius R_0 in a sphere within which a Brownian walker can be found initially with uniform probability. The $N = 12$ red circles on the surface of the sphere represent the absorbing targets.

To evaluate Eq. (3.3) one needs to determine the functional dependence of the splitting probabilities $P_i(r,\theta)$ in polar coordinates, where the subscript $i = 1,...,N$ indicates the individual targets on the disk boundary. In other words one needs to compute the following N

integrals

$$\langle P_i \rangle_{\text{disk}} = \frac{1}{\pi R_0^2} \int_0^{R_0} \int_0^{2\pi} P_i(r, \theta)\, r \,\mathrm{d}\theta \,\mathrm{d}r, \qquad i = 1, ..., N. \tag{3.4}$$

To proceed further in the calculation, one needs to realize that is more convenient to perform the θ integration first. In other words, it is necessary to evaluate the integral

$$\rho_i(r) = \int_0^{2\pi} P_i(r, \theta)\,\mathrm{d}\theta. \tag{3.5}$$

Due to the symmetry of the target positions, the function $\rho_i(r)$ does not depend on the subscript i at all. In this case the splitting probabilities $P_i(r, \theta)$ are just rotated copies of each other (see the example in Fig. 1b), which all have exactly the same integral over the polar angle. Hence one can replace $\rho_i(r)$ with a function $\rho(r)$ and perform a θ integration over the matricial equation (1.1) obtaining

$$M \begin{pmatrix} \rho(r) \\ : \\ \rho(r) \end{pmatrix} = \begin{pmatrix} 2\pi \\ : \\ 2\pi. \end{pmatrix}$$

For the above system to hold, the sum of every row of M must yield a value that does not depend on the row index. For targets on the boundary Eq. (1.4) shows that $H_{ij} = H_{ji}$ and that $\sum_{j=1}^{N} H_{ij} - H_{jk} = 0$ irrespective of the choice of i and k. Summing over j in Eq. (1.2) then gives the relation $\sum_{j=1}^{N} M_{ij} = N$ and therefore $\rho(r) = 2\pi/N$. As a result Eq. (3.4) reduces to a constant function

$$\langle P_i \rangle_{\text{disk}} = \frac{1}{N}, \tag{3.6}$$

independent of r.

To calculate $\langle H_{kS} \rangle = \langle H(\mathbf{r}_k|\mathbf{r}) \rangle$, where in polar coordinates $\mathbf{r} = (r, \theta)$ is the starting position of the Brownian particle, and $\mathbf{r}_k = (1, \theta_k)$ is the coordinate of the midpoint of an absorbing target, one first determines from Eq. (1.4) that

$$H(1, \theta_k | r, \theta) = -\frac{\ln[1 + r^2 - 2r\cos(\theta - \theta_k)]}{\pi} + \frac{r^2}{4\pi} - \frac{1}{8\pi}. \tag{3.7}$$

The intergation $\int_0^{R_0} \int_0^{2\pi} H(1, \theta_k | r, \theta)\, r\,\mathrm{d}\theta\,\mathrm{d}r$ can now be done analytically, providing the result

$$\langle H_{kS} \rangle = \frac{R_0^2 - 1}{8\pi}, \tag{3.8}$$

which is valid for a radius R_0 of the region where initially an effector is located. The value of the radius is such that it does not overlap with the region of absorption of the targets, that is $R_0 < 1$. In the main text for some of the figures, we have taken $\langle H_{kS} \rangle = 0$ in the limit $R_0 \to 1$.

Finally, by using Eqs. (1.4) and (1.5) in (1.8) and the result in (3.8), the GMFPT expression in Eq. (3.2) simplifies to

$$\langle T \rangle_{\text{disk}} = \frac{1}{DN} \left[-\ln(\varepsilon) + \frac{1}{8} + \sum_{k=1}^{N-1} \frac{N-k}{N} \left\{ \ln\left[2 - 2\cos\left(\frac{2\pi k}{N}\right) \right] + \frac{1}{4} \right\} + N\frac{(1 - R_0^2)}{8} \right], \tag{3.9}$$

which is valid if the absorbing targets are placed symmetrically along the disk boundary.

3.2 Spatial averaging in 3d

For the unit sphere, we consider the region where effectors may be located as a concentric sphere A with dimensionless radius R_0 (see Fig. 4b). In this case the spatial average in Eq. (3.1) is given by

$$\langle T \rangle_{\text{sphere}} = \frac{3}{4\pi R_0^3} \int_0^{R_0} \int_0^{2\pi} \int_0^{\pi} T(r, \phi, \theta)\, r^2 \sin(\theta)\, \mathrm{d}\theta\, \mathrm{d}\phi\, \mathrm{d}r, \tag{3.10}$$

where the integral is performed over the spherical coordinates (r, θ, ϕ).

When the absorbing targets are placed in a symmetric fashion on the unit sphere, Eq. 3.10 can be simplified. One example is displayed in Fig. 4b whereby 'symmetric' means the following: imagine painting one of the absorbing targets white, and the other ones blue. Now repeat this procedure, but for a different white target. Can we rotate the second picture (with the new white target) in such a way that the first picture is recovered? If so, then the splitting probability functions $P_i(\mathbf{r})$ are simply rotated copies of one another. The average of these functions over a full solid angle (performing the two angular integrals in Eq. (3.10)) will be the same for all target labels i. Hence, the integral

$$\rho_i(r) = \int_0^{2\pi} \int_0^{\pi} P_i(r, \phi, \theta) \sin(\theta)\, \mathrm{d}\theta \mathrm{d}\phi \tag{3.11}$$

does not depend on the index i and we call it $\rho(r)$.

As for the 2d case, using this symmetry we can integrate over a full solid angle the matricial equation (1.1) and obtain

$$M \begin{pmatrix} \rho(r) \\ \vdots \\ \rho(r) \end{pmatrix} = \begin{pmatrix} 4\pi \\ \vdots \\ 4\pi \end{pmatrix}.$$

This system of equations holds only if the sum of each row of the matrix M gives the same constant value. From Eq. (1.6) one can show that for targets on the boundary

$$H_{ij} = \frac{1}{2\pi\sqrt{2(1 - f_{ij})}} + \frac{1}{4\pi} \ln \left[\frac{2}{1 - f_{ij} + \sqrt{2(1 - f_{ij})}} \right] - \frac{9}{20\pi}, \tag{3.12}$$

with

$$f_{ij} = \cos(\theta_i)\cos(\theta_j) + \sin(\theta_i)\sin(\theta_j)\cos(\phi_i - \phi_j), \tag{3.13}$$

which shows explicitly that $H_{ij} = H_{ji}$ and thus $\sum_{j=1}^{N} M_{ij} = N$. We can then conclude, as for the 2d case, that $\rho(r) = 4\pi/N$, which gives

$$\langle P_i \rangle_{\text{sphere}} = \frac{1}{N}. \tag{3.14}$$

To find $\langle H_{kS} \rangle$ one needs to evaluate the following integral

$$\langle H_{kS} \rangle_{\text{sphere}} = \frac{3}{4\pi R_0^3} \int_0^{R_0} \int_0^{2\pi} \int_0^{\pi} H(\mathbf{r}_k | r, \phi, \theta)\, r^2 \sin(\theta)\, \mathrm{d}\theta\, \mathrm{d}\phi\, \mathrm{d}r, \tag{3.15}$$

where $H(\mathbf{r}_k|r,\phi,\theta)$ can be determined from Eq. (1.6). The symmetry of the problem is such that does not depend on the index $\mathbf{r}_k$. For convenience we consider $\mathbf{r}_k$ along the vertical axis, such that all the dot-products in (1.6) depend on the polar angle θ in a straightforward manner. The integrand in Eq. (3.15) is thus

$$\begin{aligned} H(\mathbf{r}_k|r,\phi,\theta) &= \frac{1}{2\pi\sqrt{1+r^2-2r\cos(\theta)}} + \frac{1}{4\pi}\ln\left[\frac{2}{1-r\cos(\theta)+\sqrt{1+r^2-2r\cos(\theta)}}\right] \\ &\quad + \frac{r^2}{8\pi} - \frac{23}{40\pi}. \end{aligned} \tag{3.16}$$

The integration in spherical coordinates of the logarithmic term gives exactly zero and the remaining integrals give

$$\langle H_{kS}\rangle_{\text{sphere}} = \frac{3}{40\pi}(R_0^2-1), \tag{3.17}$$

which is valid for a radius R_0 of the region where initially an effector is located. As for the 2d case, the value of the radius is such that it does not overlap with the region of absorption of the targets, that is $R_0 < 1$. In the main text for some of the figures, we have taken $\langle H_{kS}\rangle = 0$ in the limit $R_0 \to 1$.

By using Eqs. (1.6) and (1.7) in (1.8) and the result in (3.17), the GMFPT in Eq. (3.2) for symmetrically placed targets can be reduced to the expression

$$\langle T\rangle_{\text{sphere}} = \frac{3\pi}{4D}\left[\frac{1}{N^2}\sum_{k=1}^{N}\sum_{j=1}^{N} H_{jk} - \frac{3}{40\pi}(R_0^2-1)\right]. \tag{3.18}$$

Finally, to recover Eq. (2.3) of the main text, it is sufficient to split the double summation in Eq. (3.18) into a sum containing the terms with $j = k$ and those containing $j \neq k$.

References

[1] G. Barton, *Elements of green's functions and propagation*, first ed., Oxford University Press, 1989.

[2] C. Chevalier, O. Bénichou, B. Meyer, and R. Voituriez, *First-passage quantities of brownian motion in a bounded domain with multiple targets: a unified approach*, J. Phys. A: Math. Theor. **44** (2011), 025002.
